# Advancing the Cardiovascular Workforce in Africa to Tackle the Epidemic of Cardiovascular Disease: The Time is Now

**DOI:** 10.5334/gh.1197

**Published:** 2023-04-21

**Authors:** Dzifa Ahadzi, Bamba Gaye, Yvonne Commodore-Mensah

**Affiliations:** 1 Department of Medicine, Tamale Teaching Hospital, Tamale, Ghana; 2 INSERM, U970, Paris Cardiovascular Research Center, Department of Epidemiology, Paris, France; 3 Université de Paris, INSERM, Paris Cardiovascular Research Centre, Paris, France; 4 Johns Hopkins University School of Nursing, Baltimore, Maryland, USA

**Keywords:** Africa, cardiovascular disease, health workforce, health systems

## Abstract

The African region is experiencing an epidemic of cardiovascular disease with dire consequences of increasing morbidity and mortality. Compared with high-income countries where older populations are most affected, the burden of CVD in Africa is higher in the younger populations, which hampers regional socioeconomic development. Strategies to increase and advance the cardiovascular workforce are urgently needed to help address this problem. This commentary highlights the critical lack of skilled cardiovascular healthcare professionals, including cardiologists, cardiac surgeons, and cardiovascular nurses in the African region. Multilevel viable solutions to advance the cardiovascular workforce in Africa based on successful models in Africa are also presented.

Cardiovascular disease (CVD) is an important cause of hospitalization and premature death in Africa [1]. In Africa, CVDs form the majority of noncommunicable diseases (NCDs) and account for an estimated 13% of deaths and 37% of NCD-related deaths [1]. Though NCDs are currently the second most common cause of death in Africa, it is predicted that they will become the leading cause by 2030 and account for over 50% of deaths [2]. Over one million CVD-related deaths were recorded in Africa in 2019 [3]. Africa’s CVD epidemic is primarily driven by hypertension, with ominous outcomes of strokes and heart diseases, such as hypertensive heart disease and ischemic heart disease [4]. With rapid urbanization in Africa, other CVD risk factors, such as obesity, physical inactivity, hyperlipidemia, smoking and diabetes, are also on the rise [1]. Moreover, the African region has significant gaps in cardiovascular care, which is reflected in the rising burden of CVD despite its preventable nature. Also concerning is the occurrence of CVD at younger ages in Africa compared with high-income countries (HICs) [4], which may stifle the ongoing sustainable development goal of economic growth in the African region.

In order to tackle the growing burden of CVD in Africa, it is important to consider the unique dynamics of Africa’s CVD epidemic. Identifying environmental exposures and novel disease mechanisms, such epigenetic modifiers, can help discover a set of therapeutic targets useful for directed screening and ultimately achieving better treatment for every individual [5]. In addition, robust health systems equipped with the essential logistics to provide CVD care across all spectrums of disease should be built or enhanced. According to the World Health Organization (WHO), a well-functioning health system is characterized by trained healthcare workers (HCWs), a well-maintained infrastructure, reliable medication supplies and technologies, appropriate healthcare financing and evidence-based policies [6]. However, a strong health workforce is the foundation of a well-functioning health system [6].

Though there are shortages of HCWs globally, Africa is severely affected [6]. Although Africa has 24% of the world’s disease burden, it has only 3% of HCWs globally [7]. Considering population size, WHO estimates that Africa has one of the largest need-based shortages of HCWs at 4.2 million, with a projected increase to 6.1 million by 2030 [6]. WHO recommends a minimum density threshold of 2.28 doctors, nurses, and midwives per 1,000 population to achieve universal health coverage [6]. Out of 47 countries in the African region, only 9 countries attained or exceeded this standard in 2018 [6]. In the same year, almost 50% of countries in the African region had a health workforce density per 1,000 population of less than 1.0 doctors, nurses and midwives [6]. In 2018, physicians per 1,000 population amounted to 0.31, while nurses and midwives per 1,000 population amounted to 1.23, reflecting insufficient increases over the past decade and highlighting the urgent need for Africa to expand its health workforce [6].

Approximately 1% of COVID-19-related HCW deaths occurred in the African region, with indications that the figure may be higher due to underreporting [8]. Although the actual COVID-19 death toll among HCWs in the African region is unknown, it is plausible that the pandemic has further decimated the limited health workforce in Africa. There has also been a significant reduction in the health workforce in Africa due to international migration in recent years, which warrants urgent attention [6]. Since financial security and the desire for professional development are key motivations for HCW migration [9], interventions to retain HCWs must address these factors.

A key objective of the African regional framework for the implementation of the Global Strategy on Human Resources for Health developed in 2017 was to align investment in human resources for health with current and future population and health system needs [6]. Given the increasing burden of CVD in Africa and the projected impact of this problem, CVD care should be a top priority. The cardiovascular workforce represents a critical mass that needs to be increased to help address this problem.

Healthcare systems in the African region are constrained due to the double burden of infectious diseases and NCDs compounded with a shortage of HCWs compared with HICs [10]. The availability of human resources for cardiovascular care is limited in the African region, and few HCWs are trained and experienced in managing CVDs [10,11]. A lack of training facilities in some countries and the long training duration mean that Africa may not be able to produce enough cardiologists to manage the population’s cardiovascular needs in the near future [1,12]. A survey conducted in 2017 revealed that 18% of 33 participating African countries did not have a registered cardiologist [13]. Africa has about 2,000 cardiologists for a population of 1.2 billion, with a disparate distribution across countries [12]. In addition, there is limited availability of interventional and cardiac surgery services, with only 22 cardiothoracic centers in Africa [11,12]. Also, 30% of African countries do not have consistent access to invasive therapies (i.e., pacing and catheter ablation) [14]. In most African countries, interventional services are provided through collaborations with cardiologists from HICs [1]. Furthermore, many of these services are available almost exclusively in urban areas, which leads to significant inequity and disparities in care in rural areas [11].

Task-shifting, as a component of team-based care, describes the allocation of healthcare tasks usually performed by physicians to other HCWs [10] and is a viable strategy recommended by WHO for addressing the critical shortages in the health workforce [6]. Task-shifting is recognized as a useful model for HIV, maternal and child care and has been adopted for cardiovascular care with success in some African countries [6,10]. Successful task shifting models for CVD care have been implemented in low- and middle-income countries (LMICs) where community health workers, nurses, and pharmacists were trained to manage CVD [10]. In these models, individuals with a moderate to high CVD risk were linked to physicians for comprehensive evaluation and treatment [10]. For secondary prevention, non-physicians can assist with distributing medications, monitoring adherence and offering support for self-care, similar to the HIV and tuberculosis program care models [10]. For successful implementation, team-based care requires clear guidelines, continued training, supervision, physician support, prescription privileges in some cases and a commitment to financing of essential logistics [10].

As training cardiovascular healthcare professionals requires a long duration of time, global, regional and local education and skills training can contribute to rapidly increasing the number of skilled cardiovascular workers in Africa. Models that foster skills transfer within existing infrastructure in African countries have previously been reported and found to be effective [11,12]. These include short courses in pacemaker implantation for physicians [12] and long-term collaborative partnerships with resource-equipped centers to build local expertise in interventional cardiology and cardiac surgery [11,14].

This concept is exemplified in the model of the Aswan Heart Centre in Cairo, Egypt, one of Africa’s largest cardiovascular care centers, which was established almost two decades ago by a renowned British-trained Egyptian cardiac surgeon, Professor Sir Magdi Yacoub. The model involved financial backing from a local heart foundation to provide modern infrastructure, a robust translational research program and international collaboration through intermittent visits of doctors, nurses and technicians to build local expertise. With time, dedicated and persevering local staff maintained continuous operations to hone their skills, even in the absence of visiting professionals from HICs. As a result, the center has seen a dramatic scale up in its workforce, with skills now being transferred to local trainees by locally trained staff. This model represents a feasible approach to ensuring skills transfer through international collaborations [15].

Echocardiography services can be expanded by skills training in focused echocardiography for non-cardiologists to facilitate prompt diagnosis and treatment of CVDs. With this approach, obstetricians have been trained to perform screening echocardiography on pregnant women to relieve the workload of cardiologists in a South African institution [12]. The numbers of cardiovascular nurses, a critical part of the CVD workforce, can be increased through sustainable local and global partnerships to address the growing burden of heart failure and other CVDs. In Rwanda, nurses were successfully trained to perform focused echocardiography as part of their duties in running nurse-led district heart failure clinics in a long-term partnership with an international health organization [16]. As nurse autonomy in frontline CVD care increases, nurse-physician barriers must be simultaneously addressed to facilitate the acceptability and sustainability of this strategy [17].

Simplified cardiovascular guidelines, protocols and algorithms for the management of CVD can guide non-cardiologists to institute appropriate cardiovascular care across all levels of the healthcare system. Clinical guidelines are important tools because they provide a summary of evidence-based approaches to patient care [18]. Only 65% of African countries had guidelines for CVD management in 2013, and 63% of these countries had distributed the guidelines and made them readily available [18]. In healthcare settings where clinical guidelines are available, they are often poorly operationalized and underutilized by healthcare professionals [7]. Unfortunately, most CVD guidelines in Africa lag behind current global standards of care and heavily rely on the evidence-base from HICs [18]. To bridge this care gap, relevant stakeholders, such as ministries of health, national CVD organizations and clinicians with expertise in CVD management, must collaborate to develop and widely disseminate current evidence-based context-appropriate clinical guidelines.

There is scarce data on CVDs in Africa, which may be responsible for the African region’s slow response to this ongoing epidemic [11]. The World Heart Observatory, a global resource launched by the World Heart Federation to provide actionable data to curb the burden of CVD, has shown critical gaps in data from the African region [19]. Policy makers need access to timely and accurate data on the burden of CVD in Africa to inform the restructuring of health systems to address this issue. Such data would inform the formulation of strategies to advance Africa’s cardiovascular workforce. The Academic Model Providing Access to Healthcare (AMPATH) Consortium represents a model of an academic partnership that strengthened the research skills of African-based researchers with the additional benefit of improving the cardiovascular health of the local populace [20]. Through the long-term partnership of Duke University and a public hospital in Kenya, local cardiology fellows gained research training and conducted projects that addressed locally relevant cardiovascular problems with positive effects on cardiovascular care delivery [20]. There is a pressing need for adequate funding from local and international funding agencies for research that will capture the real magnitude of CVD in Africa as a basis for designing multilevel interventions to curb this epidemic.

In conclusion, strategies to advance Africa’s cardiovascular workforce are urgently needed to tackle the current and projected increase in CVD burden. In this regard, team-based care, partnerships for education and skills training, the development of evidence-based contextually appropriate CVD guidelines and academic partnerships for relevant CVD research may be promising approaches. Considering the enormity of this task, it will require the joint effort and commitment of the local community, patients, and national and international actors, including politicians, scientists, clinicians, and nongovernmental organizations.
